# Paroxetine—Overview of the Molecular Mechanisms of Action

**DOI:** 10.3390/ijms22041662

**Published:** 2021-02-07

**Authors:** Magdalena Kowalska, Jacek Nowaczyk, Łukasz Fijałkowski, Alicja Nowaczyk

**Affiliations:** 1 Department of Organic Chemistry, Faculty of Pharmacy, Ludwik Rydygier Collegium Medicum in Bydgoszcz, Nicolaus Copernicus University in Toruń, 2 dr. A. Jurasza St., 85-094 Bydgoszcz, Poland; magda.kowalska@doktorant.umk.pl; 2 Faculty of Chemistry, Nicolaus Copernicus University in Toruń, 7 Gagarina Str., 87-100 Torun, Poland; jacek.nowaczyk@umk.pl

**Keywords:** paroxetine, CYP, MAT, GRK2, EBOV

## Abstract

In the 21st century and especially during a pandemic, the diagnosis and treatment of depression is an essential part of the daily practice of many family doctors. It mainly affects patients in the age category 15–44 years, regardless of gender. Anxiety disorders are often diagnosed in children and adolescents. Social phobias can account for up to 13% of these diagnoses. Social anxiety manifests itself in fear of negative social assessment and humiliation, which disrupts the quality of social functioning. Treatment of the above-mentioned disorders is based on psychotherapy and pharmacotherapy. Serious side effects or mortality from antidepressant drug overdose are currently rare. Recent studies indicate that paroxetine (ATC code: N06AB), belonging to the selective serotonin reuptake inhibitors, has promising therapeutic effects and is used off-label in children and adolescents. The purpose of this review is to describe the interaction of paroxetine with several molecular targets in various points of view including the basic chemical and pharmaceutical properties. The central point of the review is focused on the pharmacodynamic analysis based on the molecular mechanism of binding paroxetine to various therapeutic targets.

## 1. Introduction

Anxiety is an emotional state characterized by feelings of unreasonable fears, feelings of danger, and may have different severity and duration. Once anxiety exceeds personal adaptive abilities, its intensity and duration may be disproportional in relation to the stimulus that has triggered it. This sense of fear is modulated by some regions of the brain such as the amygdala, hippocampus and prefrontal cortex. Incorrect adjustments in the tuning of specific circuit components, including deficiencies in the dampening of amygdala stress responses by prefrontal regions, are involved in alterations in fear response. Clinical anxiety often causes intense need to escape, which may result in immediate relief from symptoms. Such avoidance is so reinforcing that it can quickly become a habit that creates increasingly impaired functioning. Fear is associated with the occurrence of somatization symptoms (rapid heart rate, sweating, tremor, dyspnea, fast breathing) due to vegetative imbalance leading to significant decline in daily functioning [1].

Anxiety disorders (ADs, ICD-10-CM code: F40-F48, [2]) are reported to be the most common mental disorders worldwide. In the USA, they affect 18 percent of the general population, which is more than twice as much as mood disorders (including recurrent depression and bipolar disorder) and twenty-fold more than schizophrenia [3]. The prevalence of ADs in children and adolescents is estimated to be in range from 6 to 20%, placing these diseases among the most common mental disorder/illnesses in developed countries. According to the American Society of Anxiety and Depression (ADAA) estimates, only a third of patients suffering from AD receive adequate help and treatment despite the fact that ADs are highly treatable. In children, before the age of 12, separation anxiety is the most common, and its occurrence decreases with age [3,4]. On the contrary, the most common AD among adolescents is social anxiety disorder (SAD, ICD-10-CM code: F40.1, F40.10, F40.11 [5]) (0.3–13.1%), the spread of which increases with age. The peak incidence occurs at adolescence and early adulthood. Throughout the lifespan, females are more likely to suffer from AD (9.5%) than males (4.9%) [4].

SAD, also called social phobia, is the second most common AD and is characterized by fear of public assessment and humiliation. The disorder leads to significant disturbances in social functioning of a patient [6]. Despite the availability of effective treatments, fewer than 5% of people suffering from SAD seek professional help during the first year after the initial onset [3,7,8].

Treatment of the above-mentioned disorders is based on psychotherapy and pharmacotherapy. In case of child and adolescent patients, only the former is approved as the first line. However, recently, it often happens that pharmacotherapy is introduced using some unapproved substances. Prescription outside the indications occurs when a child receives a drug that has not been approved by the appropriate agency (e.g., FDA or EMA) for a given diagnosis or child patient age. This so-called off-label prescribing frequently occurs despite the lack of information on medication safety, efficacy and proper use in children (e.g., dosing and interactions). Furthermore, off-label prescribing has been associated with adverse drug events [9]. 

Paroxetine (ATC code: N06AB [10]; DrugBank ID: DB00715 [11]) belongs to the SSRI group (selective serotonin reuptake inhibitors,) and according to the literature, it is one of the most common off-label drugs used in daily clinical practice [12]. The plethora of publications devoted to the drug deliver a variety of information concerning different aspects of the drug’s use and modes of action. Nevertheless, there is a lack of papers devoted to explanation of paroxetine’s mechanism of action on specific targets based on X-ray-supported structural studies. Unapproved prescription of medicines to children has been rated to fall in the range between 11 and 79% worldwide. Serious side effects or mortality from antidepressant drugs overdose are currently rare [13]. Paroxetine was estimated to be among the five most commonly used drugs for depression in children [14]. Recent studies indicate that paroxetine has promising therapeutic effects and is used off-label in children and adolescents [15]. In a natural way, these facts clearly indicate the need to discuss the possibility of using paroxetine as indicated for young patients. For this reason, the main issue of this work is the presentation of data related to the description of paroxetine activities tested at the molecular level based on the collected crystallographic data.

The purpose of this review is focused on description of the interaction of paroxetine with several molecular targets from various perspectives. In the paper, the basic chemical and pharmaceutical properties of paroxetine are discussed to provide a proper foundation for further discussion. Later in the text, detailed pharmacodynamic analyses are drawn based on the molecular mechanism of paroxetine’s binding to all available therapeutic targets that have structure confirmed by X-ray studies. Binding of a ligand to a specific target protein requires a specific arrangement of both the ligand and binding site in the protein. The attractive interactions between ligand and target usually have the nature of noncarbonated contacts (hydrogen bonding, Van der Waals interactions and so on). Detailed characterization of the interaction patterns between paroxetine and analyzed proteins was carried out in the course of this study. For each biological activity discussed here, a detailed 2D representation of protein–ligand interactions is presented using LigPlot+ software [16]. A uniform method of visualization was adopted to better represent the interaction for all the therapeutic targets analyzed.

## 2. Paroxetine—General Information and History

Paroxetine is a drug indicated for the treatment of variety of anxiety disorders, including generalized anxiety disorder (GAD, ICD-10-CM code: F41.1 [17]), obsessive–compulsive disorder (OCD, ICD-10-CM code: F42.3, F42.2, F42.9, F42.8 [18]), major depressive disorder (MDD, ICD-10-CM code: F32, F33 [19]), premenstrual dysphoric disorder (PDD, ICD-10-CM code: F32.81 [20]), post-traumatic stress disorder (PTSD, ICD-10-CM code:F43.1 [21]), panic disorder (PD, ICD-10-CM code: F41.0 [22]), social anxiety disorder (SAD, ICD-10-CM code: F40.1, F40.10, F40.11 [5]) and vasomotor symptoms [23,24,25,26]. It is worth emphasizing that in the treatment of PTSD there are only two approved pharmacotherapies based on SSRIs, including Paxil (paroxetine hydrochloride). In all of the above disorders, pharmacotherapy is used in children and adolescents with off-label markings. First-line treatments for depression among children are nonpharmacological approaches [13,27].

Preliminary evidence indicates better therapeutic efficacy of paroxetine in children and adolescents with OCD, social phobia or depression when compared to that in adults [28]. However, there have been reports in the literature indicating the influence of paroxetine therapy in children and adolescents suffering from severe depressive disorders on the increase of suicidal tendencies, as compared to placebo [29,30,31,32,33,34,35,36,37]. For this reason, no antidepressants, including paroxetine, are contraindicated in children [34,38,39]. Antidepressants are used to treat depression and prevent disease-induced suicide, the possibility that drug-induced suicide can appear as side effect is a serious issue that needs to be thoroughly investigated [27,38,39,40,41]. Existing studies on the link between suicide and antidepressants vary with different results and continue to cause a lot of controversy [32,33,34,35,36]. In addition, it is estimated that the risk of negative effects of untreated or undertreated depression is usually higher than the risk of drug-induced suicide [27,41].

Paroxetine is an active substance of drugs known by the trade names Aropax, Paxil, Pexeva, Seroxat, Sereupin and Brisdelle. It was first marketed in the U.S. in 1992 under the proprietary name Paxil [42]. It is administered orally as a solid dose tablet, oral suspension, or controlled-release tablet [43]. In its clinical efficacy paroxetine can be compared with tricyclic antidepressants; however, it is safer and has greater acceptance by the patients [44,45]. According to the information provided by Paxil manufacturer GlaxoSmithKline and approved by the FDA, the effectiveness of this drug in MDD has been proven by six placebo-controlled clinical trials. For panic disorder, three 10–12-week studies indicated paroxetine’s superiority to placebo. Similarly, three 12-week trials for adult outpatients with social anxiety disorder demonstrated better response to paroxetine than to placebo [46,47,48]. It has also been used in the treatment of diabetic neuropathy, vasovagal syncope and chronic headache [49]. Paroxetine also has proven effective in the treatment of vasomotor symptoms (e.g., hot flashes, night sweats) in women undergoing menopausal transition and in patients receiving antiestrogenic cancer therapy [50]. Paroxetine is also used as a veterinarian medicine. It has been proven useful in the treatment of canine aggression and stereotyped or another OCD behavior. It has also been used in cats from time to time [51]. 

In pharmacological studies, various tests were conducted to confirm the expected biological activity, e.g., for serotonin transporter (SERT) inhibition or to test a specific mechanism of action as is the case in *Ebolavirus* (EBOV) studies. There has also been an accidental discovery of unexpected activity towards disorders in the circulatory system. Table 1 summarizes the results of crystallographic paroxetine studies from different perspectives.

Facts related to therapeutic costs cannot be ignored as well. According to GlobalData Projects Drug estimates increase of sales for PTSD in the seven major markets (7MM: the US, France, Germany, Italy, Spain, the UK and Japan) from $211.4 million in 2018 to $1.2 billion in 2028, at a CAGR of 18.7% [60]. The cited data demonstrate the scale of this issue. Nevertheless, this thread is beyond the scope of the present review.

## 3. Paroxetine—Chemistry

**Figure 1:**
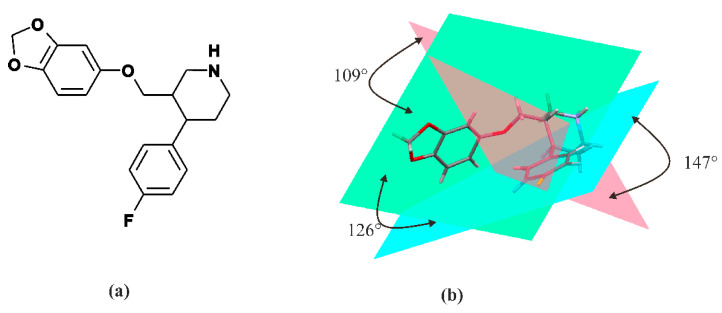
Molecular structure of paroxetine: 2D semi-structural scheme (**a**) and 3D stick representation with indicated planes of rings. The pink plane corresponds to the piperidine ring, green one to the benzodioxol ring and blue one to the fluorophenyl ring (**b**).

Paroxetine is a phenylpiperidine derivative. It is composed of a secondary amine residing in the piperidine ring, which in turn is connected to benzodioxol and fluorophenyl groups [54] (Figure 1). From a chemical point of view, paroxetine is enantiomerically pure, (−)-(3S,4R)-3-[(2H-1,3-benzodioxol-5-yloxy)methyl]-4-(4-fluorophenyl)piperidine hydrochloride hemihydrate with empirical formula of C_19_H_20_FNO_3_·HCl·½H_2_O (PubChem CID: 43815 [61]). It is an odorless, off-white powder, having a melting point ranging between 120 and 138 °C. Particularly, paroxetine is a relatively small molecule with molecular weight of 374.8 g/mol (329.4 g/mol as free base). [47]. In addition to hydrochloride, paroxetine mesylate is also available [23]. It can be concluded that nowadays, the structure of paroxetine is well-researched and understood (Figure 1). The compound exists in two crystal forms, i.e., a stable hemihydrate referred to as form I and the anhydrous form called form II (CCDC DN: 125003 [62]) [63,64]. Spectroscopic data are available in the literature and databases: FTIR (SpectraBase Compound ID: 31iWZ0aC88k [65]) NMR ([66]) MS (accession: AU152606 [67]). These data clearly show that each of the three rings present in paroxetine structure is located on a different plane, so the structure of this compound is a highly nonplanar molecule, as is depicted in Figure 1.

It is a lipophilic base amine with both hydrophobic and hydrophilic moieties (pKa is 9.9 and the partition coefficient of paroxetine (log Po/w = 3.95)) [44]. It is slightly soluble in H_2_O (5.4 mg/mL), sparingly soluble in Me_2_Cl_2_ and EtOH (96%) but entirely soluble in MeOH. The chemical properties of this compound make it easy to modify and preserve drug-like properties [43].

## 4. Paroxetine—Pharmacodynamics Pharmacokinetics and Metabolism

Paroxetine hydrochloride salt ingested orally is almost completely absorbed, with only 2% of dose recovered in feces. Biological availability of commercially available paroxetine as a controlled-release formulation (CR) is distinct. Absorption of paroxetine was found to be insusceptible to influence of food or concomitant antacid treatment. Saturation during pass through liver leads to greater bioavailability. With repeated administration the steady-state concentration of the drug is achieved within 4 to 14 days. There is no further accumulation of the compound. The distribution of paroxetine in the body is extensive and consistent with its lipophilic amine character, with only 1% of the drug remaining in systemic circulation [68,69].

The volume of distribution varies from 3,1 to 28,0 L/kg (after intravenous administration). Mean elimination half-life is estimated to be about 21 h. Almost two-thirds of the drug are eliminated through the kidneys [43,70]. Paroxetine interacts with both cytochrome P450 family 2 subfamily C member 19 (CYP2C19) and cytochrome P450 family 3 subfamily A member 4 (CYP3A4). It is also one of the most potent inhibitors of cytochrome P450 family 2 subfamily B and D member 6 CYP2D6 and CYP2B6 among SSRIs [50]. Up to 95% of the drug is bound to proteins, mainly P-glycoprotein (P-gp), which is involved in transport through the blood–brain barrier (BBB). It is worth emphasizing that paroxetine is both a substrate and inhibitor of P-gp [50,69]. Comprehensive information about human paroxetine metabolites can be found in the Human Metabolome Database [71] under the acronym HMDB0014853 [72] and Kyoto Encyclopedia of Genes and Genomes [73] under the entry D02362 [74].

### 4.1. Paroxetine as P450 Inhibitors

Cytochromes P450 (CYPs) form the main family of enzymes capable of catalyzing oxidative biotransformation of most clinically available drugs (approx. 70–80%), hormones and other lipophilic xenobiotics. For this reason, they are of particular importance to clinical pharmacology. It is widely accepted that there are 57 functional human CYPs, of which about a dozen enzymes are responsible for the biotransformation of most xenobiotics [75,76]. By far the most important are CYP3A4/5, CYP2D6, CYP2C9, CYP1A2 and CYP2B6, which metabolize 30.2%, 20%, 12.8%, 8.9% and 7.2% of currently used drugs, respectively. It has been proven that despite wide and overlapping substrate specificity of these enzymes, many drugs are metabolized at clinically relevant concentrations by only one or more enzymes, which limits the significant redundancy of the oxidation drugs system of phase I [77]. Many issues of great importance in drug treatment determine the metabolism of drugs, such as pharmacokinetics, interindividual variability and drug interactions [78]. Inhibition of metabolism mediated by P450 cytochromes often occurs as a result of interactions between different drugs. A compound that inhibits an enzyme involved in the metabolism of another drug can reduce metabolic excretion of this drug, leading to elevation of the drug’s concentration in the blood, which can cause adverse effects or enhanced therapeutic effect [79]. Paroxetine is almost completely metabolized in animals and the human system [80]. Paroxetine is well-absorbed orally and undergoes extensive first-pass metabolism that is partially saturable [43]. Its metabolites are pharmacologically inactive in vivo [81]. Paroxetine metabolism is mediated in part by CYP2D6, CYP2B6 [55,82]. 

**Figure 2:**
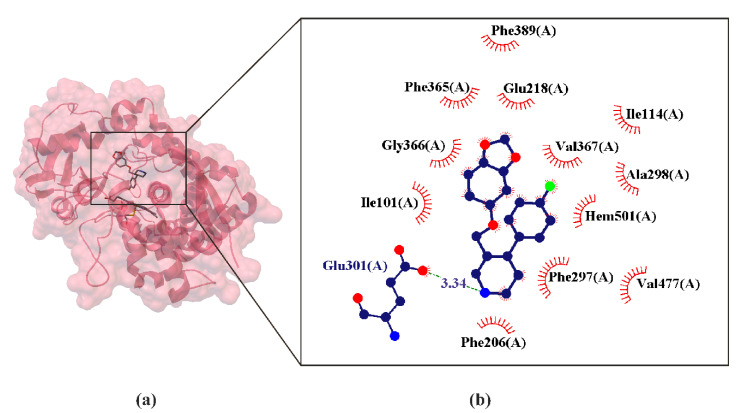
X-ray structure of paroxetine (8PR505) bind in the active site cavity of the CYP2B4 crystal (**a**), with the enlarged area showing the structural elements around the ligand-biding site (PDB ID: 4JLT, 2.14 Å) [55]. Residues that form hydrogen bonds (dashed lines) with paroxetine are shown in ball-and-stick representation with the interatomic distances shown in Å. Residues forming Van der Waals interactions with paroxetine are shown as labeled arcs with radial spokes that point toward the ligand atoms (**b**).

Numerous crystal structures have shown that all P450s share a common protein fold consisting of a large triangular prism divided into two domains. The α-helical domain (the helices are labeled in the literature A–L) with most of the helices clustered together. The smaller β-sheet domain (the sheets are labeled in the literature 1–4) (Figure 2) [83]. The first structure of a mammalian P450 was determined in 2000 [84]. According to X-ray crystal structure measurements, some P450s adopt multiple conformations, whereas other P450s appear in a single conformation. Consequently, it is difficult to predict how P450s will behave in contact with a new compound [75,76]. Active site of all P450s forms a cavity concealed within the protein molecule [85] (Figure 2). A heme coordination bond is also characteristic of each P450, combining a positively charged iron ion with a negatively charged thioplane sulphur atom [86] (Figure 2). This iron–cysteine bond is the basis of redox states which an iron ion can access during P450 catalysis, resulting in a complex catalytic cycle of P450 enzymes. In each P450, two highly synchronized electron transfer steps are required to enable the P450 catalytic cycle to reach the production stage [87] (Figure 2).

The structural studies available in the literature reveal that in the structure of paroxetine there are two possible pharmacophore points for interaction with P450: methylenedioxo moiety and secondary amine moiety of piperidine. Therefore, two possible routes of P450 inactivation by paroxetine are allowed. One involves the hydroxylation of C-H at the site of methylenedioxo moiety, and the second is N-H hydroxylation. Both C-H and N-H hydroxylation reactions can proceed via oxygen rearrangement or hydrogen atom transfer [88].

#### 4.1.1. Paroxetine as CYP2D6 Inhibitors

The CYP2D6 isoenzyme is highly polymorphic and inhibited by several small molecules and clinically important pharmaceuticals [89,90]. CYP2D6 metabolizes antidepressants, antipsychotics, analgesics, β-blockers and antiarrhythmics. Paroxetine is both a substrate and an inhibitor of cytochrome isoenzyme CYP2D6 [81]. CYP2D6 metabolizes paroxetine via demethylation of the methylenedioxy group of methylenedioxyphenol. This reaction involves oxidation of the methylene bridge to a species that forms a tight but reversible complex with the heme iron atom [91]. Paroxetine also has the highest inhibitory constant of all antidepressants for CYP2D6 (K_i_ = 0.065) [89]. Clinical drug interaction studies show that paroxetine can inhibit the metabolism of drugs metabolized by CYP2D6, such as desipramine, risperidone and atomoxetine [92]. This extremely high inhibitory binding constant or affinity explains paroxetine’s high interaction profile with substrates for CYP2D6 [93]. 

Multiple-drug therapy is a common therapeutic practice, particularly in patients with several diseases or conditions. Breast cancer is highly associated with depression and therefore requires antidepressant therapy. In animal studies, antidepressants were observed to increase the incidence and growth of breast cancer in mice. It was suggested then that it is related to the inhibition of enzymes involved in the metabolism of carcinogens and estrogens. The inhibition of the isoenzyme CYP2D6 leads to an increase in their concentrations and serum levels, which increases the risk of breast cancer [42]. Since the early 1990s, many epidemiological studies have been carried out involving women who have been using antidepressants and have been diagnosed with breast cancer. The results obtained were interpreted differently, some studies concluded that there is no association between antidepressant use and breast cancer, while others came to the entirely opposite conclusion [94]. So far, no unequivocal position has been established. Paroxetine has been shown to have estrogenic effects (mimicking estrogen at the estrogen receptors) which may potentially affect the endocrine system and the development of breast tumors in women. Nevertheless, there is no clinically valid evidence for this [95,96].

The crystal structure of CYP2D6 was resolved in 2006 at 3 Å resolution [97]. The structure of CYP2D6 has a well-defined active site cavity above the heme, the shape of which is compared in the literature to the shape of the “right foot”. The “heel” of the foot-shaped cavity lies above the heme, the foot “arch” is formed by the Phe-120 side chain, while the “ball” is bordered by residues from the B′-C loop and the N-terminal end of the I helix. Such detailed information makes it possible to use computational modelling techniques, e.g., docking in the development of drugs involving these CYPs [97]. 

#### 4.1.2. Paroxetine as CYP2B6 Inhibitors

CYP2B6 participates in the metabolism of a wide range of drug classes, including antiretrovirals, antidepressants, anesthetics, anticancer agents and antismoking agents. There is some evidence that its substrates generally contain a basic N atom and a planar aromatic ring [83]. A good example includes bupropion, cyclophosphamide, ifosfamide, pethidine, ketamine and propofol [89]. This is because this enzyme metabolizes a number of drug substrates which are usually nonplanar, neutral or weakly basic, with one or two hydrogen-bonding acceptors [98]. Paroxetine also has the highest inhibitory constant of all antidepressants for CYP2B6 (K_i_ = 1.03 μM). The inhibitory constant for CYP2D6, on the other hand, is lower compared to CYP2B6, but it is still high [93]. These high inhibitory binding constants explain paroxetine’s high interaction profile with substrates for CYP2D6 and CYP2B6 [83]. 

Paroxetine is also a potent inhibitor of CYP3A4 with multiple CYP3A4 substrate interactions. [42,99]. In humans, secondary amine xenobiotics are catalytically metabolized by P450s, leading to the formation of hydroxylamines [88]. 

#### 4.1.3. Paroxetine as CYP2B4 Inhibitors

No crystalline structure of paroxetine binds with human CYP2D6 and CYP2B6 has been obtained so far [55]. However, in the Research Collaboratory for Structural Bioinformatics (RCSB) base, the crystal of the paroxetine complex with CYB2B4 [83] is deposited. The CYP2B4 catalyst has extremely similar structure to CYP2B6, with the only difference in residue 363, which is Leu in CYP2B6 and Ile in CYP2B4. In particular, the CYP2B4 tertiary structure is the most plastic (i.e., the largest degree of structural flexibility) of any P450 studied by X-ray crystallography [100]. Since the crystal of the paroxetine complex is deposited in the RCSB base with CYB2B4, it seems reasonable to treat it as an appropriate approximation of the interaction of paroxetine with CYP2B4 as well as with CYP2B6 (Figure 2).

## 5. Paroxetine as MAT Inhibitions 

Selective serotonin reuptake inhibitors (SSRI) are clinically prescribed antidepressants that act by increasing the local concentration of the neurotransmitter at synapses and in extracellular spaces via blockade of the serotonin transporter (SERT) [53]. SERT is a member of the neurotransmitter sodium symporter (NSS) family of transporters. The NSS also includes two more catecholamine transporters such as dopamine (DAT) and norepinephrine (NET) transporters [101] and some amino acid transporters such as glycine transporter (GlyT), γ-aminobutyric acid (GABA) transporter (GAT), leucine transporter (LeuT) and osmolytes such as betaine and creatine [102,103]. Inhibitors of the three monoamine transporters (MATs) increase the extracellular concentration of monoamines, and are widely used in the treatment of psychiatric diseases and as illicit psychostimulant drugs [104]. 

Paroxetine is an SSRI that exhibits the highest known binding affinity for the central site of SERT (for SERT ≈ 70.2 ± 0.6 pM, for DAT ≈ 490 ± 20.0 nM, for NET ≈ 40 ± 0.2 nM) [105]) compared to any other currently prescribed antidepressants [54]. The selectivity profile of MAT inhibitors across NET, DAT and SERT is critical for their therapeutic profile and/or abuse potential. Binding studies have demonstrated that antidepressants are from 300 to 3500 times more selective for SERT over NET, and generally have low affinity for DAT (from 7800 to 0 times) [106]. Paroxetine acts as a dual serotonin/norepinephrine uptake inhibitor in higher doses (40 mg/day or more) [46]. Furthermore, there is evidence that paroxetine can also operate as an allosteric modulator of SERT, although not as effectively as escitalopram [107]. In vitro studies in animals suggest that it has weak effects on dopamine neuronal reuptake. It may contribute to emotional flattening, apathy and cognitive slowing in some patients. In vitro radioligand binding studies report that paroxetine has minor affinity for dopamine, alpha1-, alpha2-, beta-adrenergic-, muscarinic and histamine (H1) receptors [108,109]. Due to mild anticholinergic actions of paroxetine, the drug may cause a rapid onset of hypnotic and anxiolytic efficacy as well as the occurrence of side effects. Weak antimuscarinic properties may cause sedation, constipation and dry mouth.

**Figure 3:**
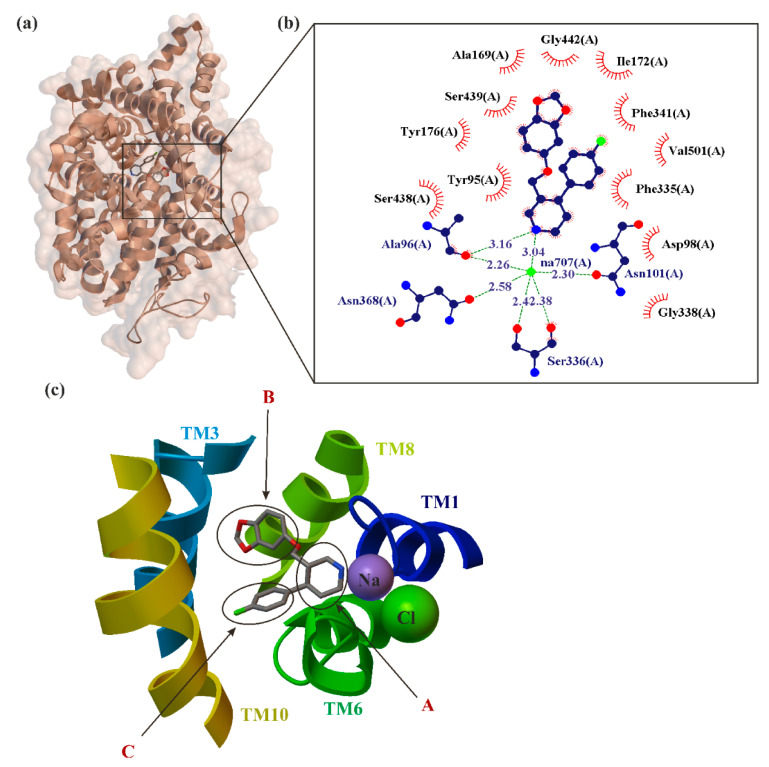
X-ray structure of paroxetine bind in the binding site of the serotonin transporter (SERT) crystal (**a**) with the enlarged area showing the structural elements around the ligand-biding site (PDB ID: 5i6x, 3.14 Å) [52]. Residues that form hydrogen bonds (dashed lines) with paroxetine are shown in ball-and-stick representation with the interatomic distances shown in Å. Residues forming Van der Waals interactions with paroxetine are shown as labeled arcs with radial spokes that point toward the ligand atoms (**b**). Schematic representation of drug interactions in the primary binding pocket of SERT (**c**) [54].

The primary target of paroxetine and other SSRI medications is the SERT, which is a type of monoamine transporter that transports serotonin from the synaptic cleft back to the presynaptic neuron [70]. SERT, as the target of many psychoactive agents, has importance to the etiology of affective disorders [52]. SERT belongs to the solute carrier 6 family of transporters (SLC6) [110]. It is an integral membrane protein that exploits pre-existing sodium, chloride and potassium ion gradients to catalyze the thermodynamically unfavorable movement of synaptic serotonin into the presynaptic neuron. The Protein Data Bank provides X-ray structures of the leucine transporter aLeuT (*Aquifex aeolicus* LeuT) [103] and monoamine transporters (MATs): dDAT (Drosophila DAT) [111] and hSERT (human SERT) [52] crystallized in 2005, 2013 and 2016. The human NET (hNET) crystal structure has not been obtained yet. SERT cotransports molecules of serotonin with one Na^+^ and Cl^−^ ions while a single potassium K^+^ ion is moved in the opposite direction. Importantly, hSERT was crystallized in complex with the two prototypical SSRIs, escitalopram and paroxetine [52] (Figure 3).

Analysis of the crystalized data amino acid sequences provided evidence for 12 transmembrane segments (TM1-TM12), with the amino and carboxy terminal end located in the extracellular vestibule. Due to high similarities to architecture of LeuT, this TM1-12 arranged often is the so-called LeuT-like structural fold. This pattern is characterized by two inverted 5-TM repeats. The location of drug-binding sites in crystallized MATs is determined according to the scheme originally determined for LeuT by Sørensen et al. [112] in two separate regions. They are marked as S1 and S2 pockets. The former is located approximately halfway across the membrane bilayer and the latter is located in the extracellular vestibule. The S1 site is the central substrate binding pocket which defines the primary binding region in NSSs, while the S2 is an allosteric site approximately 13 Å from S1. S1 is composed of three subsites, formally called A, B, C (Figure 3) [54]. From a chemical point of view, all these subsites represent different natures: A is a polar region surrounding Asp98 (side chains from TMs 1, 6 and 8), whereas subsites B and C are largely hydrophobic regions. B regions are located opposite to subsites C. B regions are formed by residues from TMs 3 and 8. Subsite C is formed by TMs 3, 6 and 10 [113]. In early 2016, the binding mode of paroxetine at the S1 SERT site was investigated simultaneously by two teams: Coleman et al. [52,53] (Figure 3) and Davis et al. [70]. The data obtained from these studies are inconclusive, the binding site and orientation of paroxetine in SERT remain controversial.

Nonplanar structure of the ligand (as in the case of paroxetine, Figure 1) causes the differences between binding poses of the ligand at the binding site obtained by different methods [114]. Piperidine, benzodioxol and fluorophenyl substituents of paroxetine were present [52,53] in subsites A, B and C of the S1 site, respectively—a pose commonly denoted by many authors as ABC (Figure 3). While the homology studies conducted by Davis et al. have proposed an orientation “flipped” from that in the ABC pose, i.e., the piperidine, benzodioxol and fluorophenyl substituents of paroxetine reside in subsites A, C, and B of the S1 site, respectively (often donated ACB) [70]. Additionally, a combination of pharmacological, biochemical and mutagenesis data suggest that amino acids implicated in high-affinity paroxetine binding may not overlap with those thought to be involved in recognizing other inhibitors [70,102].

## 6. Paroxetine as Kinase GRK2 Inhibitors 

Currently, there are five main protein families which are key therapeutic targets for most drugs available on the world market. G-protein-coupled receptors (GPCRs) are integral membrane proteins that relay external signals into the cytoplasm of the cell. GPCRs are estimated to be the main therapeutic target for about a third of prescription drugs [115,116]. They are key regulators of cell physiology and control processes ranging from glucose homeostasis to contractility of the heart. A major mechanism for the desensitization of activated GPCRs is their phosphorylation by GPCR kinases (GRKs). Overexpression of G-protein-coupled receptor kinase 2 (GRK2) is strongly linked to both the healthy and failing heart, and it has long been considered a therapeutic target for the treatment of cardiovascular disease [117]. The GRK2 originally known as β-adrenergic receptor kinase 1 (βARK-1) was the first GRK cytoplasmic protein identified in the heart [118]. It belongs to a group of serine/threonine kinases that have a relevant role in the identification and phosphorylation of activated GPCRs [119]. Accordingly, inhibition of GRK2 is considered an important drug target in the treatment of heart failure [120]. 

From a physiological point of view, the autonomic nervous system affects the frequency of heart contractions: the sympathetic system accelerates heart function while the parasympathetic system decelerates it. In response to a failing heart, the sympathetic nervous system increases the level of circulating catecholamines (norepinephrine and adrenaline). The work of the heart is also affected by β adrenergic receptors (βAR) [121]. There are four types of action β receptors on myocardial cells. These include the following effects: inotropic, chronotropic, bathmotropic, dromotropic. The first two determine the strength and frequency of myocardial contraction, the third affects the threshold of excitability of myocardial cells and the latter is responsible for the rate of conduction in the heart muscle [122]. Binding catecholamines in cardiomyocytes initiate downstream signaling to increase the contraction force of the heart muscle (i.e., inducing a positive inotropic effect) [123]. In the failing heart, activation of βARs also leads to (upregulation) increase in the number of GRK2 and GRK5, which in turn leads to uncoupling of βARs from G proteins, decreased βARs at the cellular membrane and decreased cardiac output in response to hormonal stimulation [124,125,126]. Studies of animal models have shown that reducing GRK2 levels is beneficial in preventing heart failure by renormalizing catecholamine and βAR levels of cell surfaces and improving heart function [127,128]. 

Paroxetine exhibits 50/60-fold higher selectivity for GRK2 versus other GRKs (such as GRK1 and GRK5) [56]. Paroxetine hydrochloride was identified as a modest GRK2 inhibitor with an IC_50_ of 1.4 μM [118,127]. It is worth emphasizing that other compounds from the SSRI group have shown no effect in in vitro kinase tests or ex vivo or in vivo myocyte contractility [57]. Due to this, paroxetine (or a paroxetine derivative) could be used in heart failure treatment in the future.

**Figure 4:**
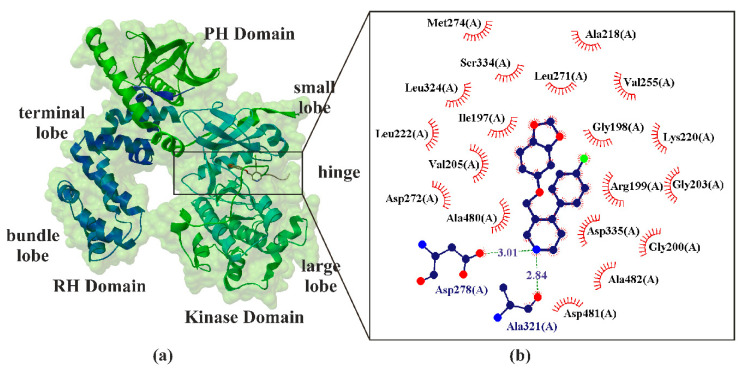
X-ray structure of paroxetine bind in the hinge region of G-protein-coupled receptor kinase 2 (GRK2) (**a**), with the enlarged area showing the structural elements around the ligand-biding site (PDB ID: 3V5W, 2.07 Å) [56,57]. Residues that form hydrogen bonds (dashed lines) with paroxetine are shown in ball-and-stick representation with the interatomic distances shown in Å. Residues forming Van der Waals interactions with paroxetine are shown as labeled arcs with radial spokes that point toward the ligand atoms (**b**).

GRK2 is a multidomain protein organized in several domains and regions (Figure 4). It consists of three distinct domains: an RGS homology (RH) domain, a protein kinase domain, and a pleckstrin homology (PH) domain. The RH domain has two subdomains often referred to as the terminal and bundle lobes, whereas the kinase domain is composed of small and large lobes. The terminal lobe of the RH domain also forms an extensive hydrophobic interface with the PH domain. The RH domain is the protein region through which GRK2 binds Gβγ. The RH domain participates in interactions with both the kinase and PH domains, thus playing an important role in the regulation of protein activity. The kinase domains containing the ATP-binding pocket consist of a β-sheet included in the small-terminal lobe (also called N-lobe) and α-helixes dominating the large-terminal lobe (also called C-lobe). Both lobes’ regions are connected via a flexible hinge region [129,130]. Most small-molecule kinase inhibitors target the ATP-binding site (Figure 4). The crystal structure of the GRK2·paroxetine−Gβγ complex revealed that paroxetine binds in the active site of GRK2 (Figure 4) and stabilizes the kinase domain in a novel conformation in which a unique regulatory loop forms part of the ligand-binding site. It was found that paroxetine inhibiting GRK2 increases contractility in isolated cardiomyocytes as well as in myocardial βAR inotropic reserve in living mice [57].

## 7. Paroxetine as *Ebolavirus* Inhibitors

EBOV belong to the family Filoviridae and cause severe, often fatal, diseases, e.g., Ebola hemorrhagic fever (EHF) in humans and other mammals, also known as Ebola virus disease (EVD, ICD-10-CM code: A98.4 [131]). EHF is characterized by rapid disease progression and high risk of death, killing between 25% and 90% of those infected, with an average of about 50% [132]. EBOV has caused the majority of human deaths from EVD and was the cause of the 2013–2016 epidemic in western Africa [133] which resulted in at least 28,646 suspected cases and 11,323 confirmed deaths [132]. There are currently no approved therapeutic drugs or vaccines for the disease [134]. 

EBOV contains single-stranded negative RNA linear genome, about 18–19 kb in size and encode 7 genes such as nucleoprotein (NP), viral protein 35 (VP35), viral protein 40 VP40, viral protein 30 VP30, viral protein 24 (VP24), polymerase (L) and glycoprotein (GP). Fatal human cases of EHF are exemplified by very high viral titers in the blood, liver and spleen, as well as profound immunosuppression [135]. 

The mechanism of EBOV inhibition is largely unknown. EBOV has a membrane envelope decorated by trimers of a glycoprotein (GP, cleaved by furin to form GP1 and GP2 subunits, Figure 2), which is solely responsible for host cell attachment, endosomal entry and membrane fusion. The trimeric transmembrane GP spike, each approximately 7–10 nm long and spaced at approximately 10 nm intervals, are presented on the surface of the virion and are accountable for cellular attachment and entry. EBOV entry into the cells is initiated by the interaction of the viral GP with receptors on the surface of host cells, and then internalized via the macropinocytosis pathway [136]. GP is thus a primary target for the development of antiviral drugs. Crystallographic studies have shown that five chemically divergent EBOV inhibitors, such as ibuprofen (pKi ≈ 2.22) [137], benztropine (pKi ≈ 2.89), bepridil (pKi ≈ 3.54), paroxetine (pKi ≈ 3.19, Figure 2) and sertraline (pKi ≈ 3.02), interact directly with the *Ebolavirus* glycoprotein [59]. Binding of these drugs destabilizes the protein, suggesting that it may be an inhibitory mechanism. The study of toremifene (anticancer drug) revealed that the binding affinities (pKi ≈ 4.80, determined by thermal shift assay) correlate with the protein−inhibitor interactions as well as with the antiviral activities determined by virus cell entry assays, supporting the hypothesis that these drugs inhibit viral entry by binding GP and destabilizing the prefusion conformation [137].

**Figure 5:**
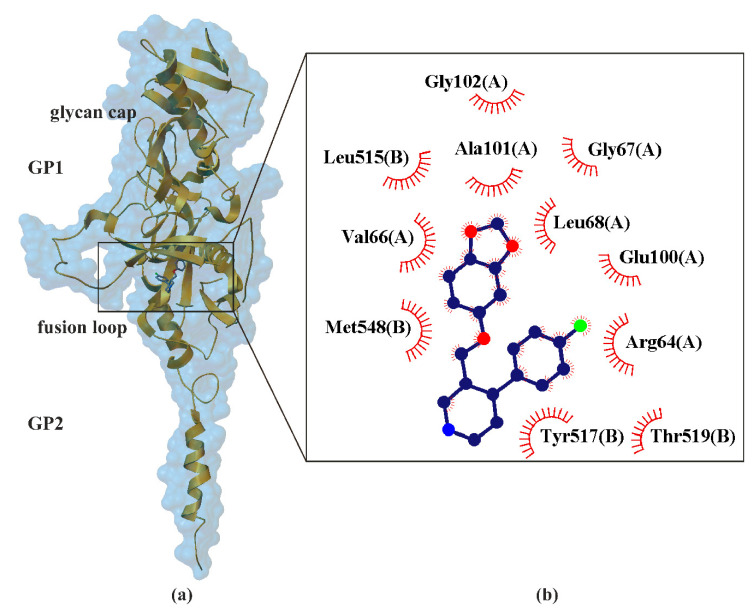
X-ray structure of paroxetine bind in the hinge region of the *Ebolavirus* (EBOV) glycoprotein (GP) (**a**), with the enlarged area showing the structural elements around the ligand-biding site (PDB ID: 6F6I, 2.40 Å) [59]. Residues that form hydrogen bonds (dashed lines) with paroxetine are shown in ball-and-stick representation with the interatomic distances shown in Å. Residues forming Van der Waals interactions with paroxetine are shown as labeled arcs with radial spokes that point toward the ligand atoms (**b**).

The crystal structure of proxetine-EBOV GP complex was determined at 2.4 Å resolution, with good R-factors and stereochemistry. GP1 has three distinct domains: (•) the receptor binding domain (RBD), (•) the glycan cap and (•) the heavily O-linked glycosylated mucin-like domain (MLD). It is predominantly composed of β-strands, forming a large semicircular groove at the center of the subunit. EBOV-GP forms GP-containing microvesicles, so-called virosomes, which are secreted from GP-expressing cells. However, determinants of GP-virosome release and their functionality are poorly understood. RBD promotes GP-virosome secretion, while tetherin suppresses GP-virosomes by interactions involving the GP-transmembrane domain [138]. RBD is responsible for interacting with one or more cellular receptors. It is proof that EBOV-GP-virosomes are immunomodulatory and act as decoys for EBOV-neutralizing antibodies. The glycan cap could protect the receptor binding sites from antibodies and interacts with the internal fusion loop of GP2 which is critical for membrane fusion. In the fusion process, GP2 undergoes conformational changes. Paroxetine binds to GP with its benzodioxol group (Figure 5) and builds interactions with not only the side chains of Val66,

Ala101, Leu515, Tyr517 and Met548, but also with the main-chains of Gly67 and Gly102 and side chain of Leu68 Figure 5 [59]. These results suggest that inhibitor binding destabilizes GP and triggers premature release of GP2, thereby preventing fusion between the viral and endosome membranes. This way of binding paroxetine from EBOV GP is analogous to those previously confirmed for other compounds, including toremifene and ibuprofen, which provides additional confirmation of the EBOV GP inhibition mechanism [137]. Given the weak inhibitory properties (low pKi) of the medicines tested so far, they are not suitable for reducing EBOV infection. Nevertheless, studies on the structures of the acquired complexes reveal the inhibition mechanism and can guide the development of more powerful anti-EBOV drugs.

## 8. Paroxetine as KIT and JAK Inhibitors 

Paroxetine can influence a variety of cancers, including brain tumor [139], colon cancer [140] and breast cancer [141] by blocking some protein kinase signaling pathways involved in tumorigenesis [142]. 

**Figure 6:**
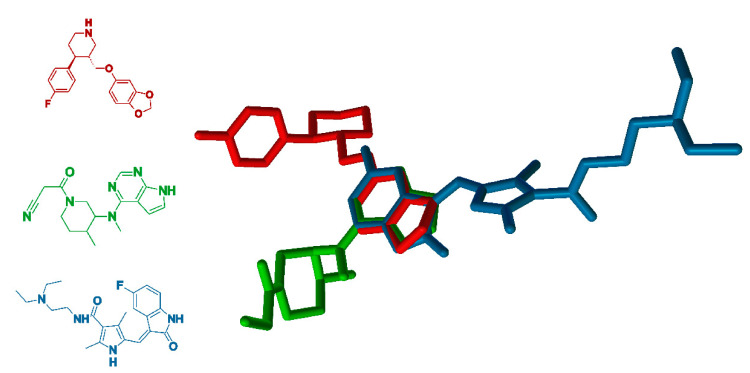
Superimposing of the paroxetine with selected tyrosine kinase (KIT) and Janus kinase (JAK) structure inhibitors: paroxetine (ATC code: N06AB05 [146], SSRI inhibitor, red), sunitinib (ATC code: L01XE04; [146] KIT inhibitor, blue) and tofacitinib (ATC code: L04AA29 [146], JAK inhibitor, green).

It has been clinically observed that paroxetine has strong cytotoxicity on human tumor cell lines. This is kinase pathways involved in tumorigenesis. Many studies have established that this is due to the structural similarities (size, shape, physicochemical properties, Figure 6) of paroxetine with many known kinase inhibitors. Consequently, it is not surprising that paroxetine is very compatible with the kinase active site [142]. A good example of paroxetine’s similarity are sunitinib, approved a multitargeted tyrosine kinase inhibitor (KIT inhibitor) [143,144] and tofacitinib (approved Janus kinase inhibitor (JAK inhibitor)) [145] (Figure 6). Sunitinib (ATC code: L01XE04 [146]), a multitargeted tyrosine kinase inhibitor, which is approved by both US and EU regulatory agencies for clinical use, extends survival of patients with metastatic renal cell carcinoma and gastrointestinal stromal tumors, but concerns have arisen about its cardiac safety [143,147,148]. Tofacitinib (ATC code: L04AA29 [146]) is a Janus kinase inhibitor currently approved for the treatment of rheumatoid arthritis, psoriatic arthritis and ulcerative colitis [145,149,150].

Tyrosine kinase proteins are a class of proteins with tyrosine kinase activity that catalyzes the transfer of phosphate groups to ATP to the residues of tyrosine of many important proteins, forming protein phosphorylation and then transmitting a signal regulating cell growth, differentiation, death and a number of physiological and biochemical processes [151]. Paroxetine can interact with these kinases in two so-called binding modes: class I and class II [142]. Five kinases in the C-Src ABL, SRC, KIT, MET and FYN family were identified as targets for paroxetine kinase, which can be inhibited strongly or moderately at IC_50_ values at the nanomolar or micromolar level. Binding modelling analysis has shown that paroxetine ligands can adopt class I binding modes to interact with KIT, MET, FYN, and class II when interacting with ABL and SRC kinases. This is possible by creating an intensive network of molecular forces: specific (i.e., hydrogen bonds and π–π/cation–π stackings) and nonspecific forces (such as hydrophobic and Van der Waals) [152] in complex kinase–inhibitor interfaces. The division into classes of bonding modes is due to the location of piperidine moieties of paroxetine at the place of active kinase. In class I, piperidine moieties of paroxetine are packed against active sit kinase. While in class II, the same molecular fragment of paroxetine adopts the opposite binding mode. It seems noteworthy that paroxetine class I binding mode shows much higher inhibition potential than that of class II [142].

## 9. Conclusions and Perspective

Recently, there has been a steadily and rapidly increasing number of prescriptions for approved and unapproved drugs for depression. This is a particularly important issue when it concerns children and young people [9]. This provides the basis for extensive research. In our opinion, we should constantly discuss and analyze current reports on drugs that are used in daily clinical practice as off-label drugs much more often than others. Indisputably, paroxetine belongs to this group of drugs. It is important to be aware that the use of off-label medicines is a multidirectional issue. Sometimes off-label use means a change in the repurpose of the drug, e.g., finding novel therapeutic indications different from the ones for which the drug was already approved [153]. This may also mean unusual use of the drug. This includes, in particular, the use of a different dose, duration of use, frequency of dosing, use of another method of administration (e.g., orally instead of intravenously) or use by another group of patients (e.g., children instead of adults) [15].

Modern scientific research in the medical and health sciences is largely based on the results of research related to the achievements of recent decades and in particular the knowledge of the genomes of both the patients and pathogens. The extraordinary dynamics of the development of this knowledge necessitate the presentation of collective reviews of recent achievements. As we have tried to show in our work, understanding the molecular aspects not only allows for a better understanding of the mechanisms of action of drugs, but also contributes to the creation of new research directions. The presented analysis of molecular studies of paroxetine based on X-ray methods seems to confirm its special place in modern pharmacotherapy. The presented results of paroxetine studies are associated with every possible aspect of studies on the use of off-label procedures. It is to be hoped that the uniqueness of the structure of paroxetine will allow the development of a new drug in heart failure, which we think would be a very valuable achievement. A number of studies of paroxetine on human cell lines of tumors confirmed its strong cytotoxicity, which generates new opportunities for the search for more drugs in oncology. On the other hand, studies on EBOV indicate the possibility of paroxetine activity among these types of pathogens. Considering the confirmed effect of paroxetine on the nervous system makes it clear that the pharmacological profile of this drug coincides with key issues of modern medicine. All this creates promising prospects. 

## Figures and Tables

**Table 1:** The list of the crystal structure of target bounded Paroxetine.

PDB ID	Target	Resolution	Organism	Reference
5I6X, 5I6Z	SERT	3.14 Å, 4.53 Å	Homo sapiens, Mus musculus	[52]
6AWN	SERT	3.62 Å	Homo sapiens, Mus musculus	[53]
6VRH	SERT	3.30 Å	Homo sapiens, Mus musculus	[54]
4JLT	P450 2B4	2.14 Å	Oryctolagus cuniculus	[55]
4L9I	GRK1	2.32 Å	Bos taurus	[56]
3V5W	GRK2	2.07 Å	Bos taurus, Homo sapiens	[57]
4MM4	LeuBAT	2.89 Å	Aquifex aeolicus VF5	[58]
6F6I	EBOV GP	2.40 Å	Ebola virus	[59]

## Data Availability

Not applicable.

## Abbreviations

AD	Anxiety Disorders
ADAA	American Society of Anxiety and Depression
ABL	Abl Tyrosine Kinases
aLeuT	*Aquifex Aeolicus* Leucine Transporter
ATC code	Anatomical Therapeutic Chemical Classification System
ATP	Adenosine Triphosphate
BBB	Blood Brain Barrier
C-Src ABL	Src Tyrosine Kinases
CYP	Cytochromes P450
CYP1A2	Cytochrome P450 Family 1 Subfamily A Member 2
CYP2B4	Cytochrome P450 Family 2 Subfamily B Member 4
CYP2B6	Cytochrome P450 Family 2 Subfamily B Member 6
CYP2C19	Cytochrome P450 Family 2 Subfamily C Member 19
CYP2C19	Cytochrome P450 Family 2 Subfamily C Member 19
CYP2D6	Cytochrome P450 Family 2 Subfamily D Member 6
CYP3A4	Cytochrome P450 Family 3 Subfamily A Member 4
CYP3A5	Cytochrome P450 Family 3 Subfamily A Member 5
DAT	Dopamine Transporter
dDAT	Drosophila Dopamine Transporter
EBOV GP	Ebolavirus Glycoprotein
EHF	Ebola Hemorrhagic Fever
EVD	Ebola Virus Disease
FDA	Food and Drug Administration
FYN	Fyn Proto-Oncogene, Non-Receptor Tyrosine Kinase
GABA	γ-Aminobutyric Acid
GAD	Generalized Anxiety Disorder
GAT	γ-Aminobutyric Acid Transporter
GlyT	Glycine Transporter
GP	Glycoprotein
GPCR	G-Protein-Coupled Receptors
GRK	G-Protein-Coupled Receptors Kinase
GRK1	G-Protein Coupled Receptor Kinase 1
GRK2	G-Protein Coupled Receptor Kinase 2
GRK5	G-Protein Coupled Receptor Kinase 5
hDAT	Human Dopamine Transporter
HEM	Hemeproteins
hNET	Human Norepinephrine Transporter
hSERT	Human Serotonin Transporter
ICD-10-CM	International Classification of Diseases, Tenth Revision, Clinical Modification
JAK	Janus Kinase
Ki	Binding Affinity
KIT	Tyrosine-Kinase Inhibitor
L	Polymerase
LeuT	Leucine Transporter
MAT	Monoamine Transporters
MDD	Major Depressive Disorder
MDL	O-linked glycosylated mucin-like domain
MET	Met Proto-Oncogene, Non-Receptor Tyrosine Kinase
NET	Norepinephrine Transporter
NP	Nucleoprotein
NSS	Neurotransmitter Sodium Symporter
OCD	Obsessive-Compulsive Disorder
PD	Panic Disorder
PDB ID	Protein Data Bank Identifier
PDD	Premenstrual Dysphoric Disorder
P-gl	P-Glycoprotein
PTSD	Posttraumatic Stress Disorder
RBD	Receptor Binding Domain
RCSB	Research Collaboratory for Structural Bioinformatics
SAD	Social Anxiety Disorder
SERT	Serotonin Transporter
SLC6	Solute Carrier 6 Family of Transporters
SRC	Src Proto-Oncogene, Non-Receptor Tyrosine Kinase
SSRI	Selective Serotonin Reuptake Inhibitors
TM	Transmembrane Segment
VP24	Viral Protein 24
VP30	Viral Protein 30
VP35	Viral Protein 35
VP40	Viral Protein 40
βARK-1	β-Adrenergic Receptor Kinase 1
